# Mapping mental health and psychosocial support service availability for adolescents and young people living with HIV in Lusaka and Shibuyunji districts, Zambia: Alignment with ART program burden (September 2024)

**DOI:** 10.1371/journal.pmen.0000610

**Published:** 2026-08-25

**Authors:** Carlos Muleya, Rosemary N. Likwa, Peter J. Chipimo, Jacqueline J. Folotiya, Ngosa Mwiya, Lweendo Mapiki, Asarf Sibuchi, Godfrey Chulu, Caitlin Baumhart, Cassidy W. Claassen, Naeem Dalal, Loyd B. Mulenga

**Affiliations:** 1 Department of Population Studies and Global Health, School of Public Health, University of Zambia, Lusaka, Zambia; 2 CAZACHA Business Solutions Limited, Lusaka, Zambia; 3 World Health Organization, Lusaka, Zambia; 4 Leaders of Africa Institute, Lusaka, Zambia; 5 Institute of Human Virology, University of Maryland School of Medicine, Baltimore, Maryland, United States of America; 6 Zambia National Public Health Institute, Lusaka, Zambia; 7 Ministry of Health, Lusaka, Zambia; PLOS: Public Library of Science, UNITED KINGDOM OF GREAT BRITAIN AND NORTHERN IRELAND

## Abstract

Adolescents and young people living with HIV (AYPLHIV) experience substantial mental health and psychosocial challenges, yet information on youth-responsive service availability is often limited for routine planning. We mapped mental health and psychosocial support services in Lusaka and Shibuyunji districts of Zambia and assessed alignment with AYPLHIV antiretroviral therapy (ART) program burden using treatment current (TX-CURR) ages 15–24 data. We conducted a cross-sectional mapping survey (10–21 February 2025) of facilities providing any mental health/psychosocial service to characterize service modalities, operating days, eligibility/consent requirements, privacy infrastructure, and ownership. We linked mapped facilities to ART sites and September 2024 TX-CURR using standardized facility names and districts, and calculated burden-weighted coverage among linked sites. As of September 2024, 16,550 AYPLHIV (15–24) were on ART across 87 sites, with 97.7% of burden in Lusaka. We mapped 88 facilities (73 Lusaka; 15 Shibuyunji); 58.0% were government-owned and 40.9% privately owned. Counseling was widely available (97.7%), whereas group therapy (31.8%), medication (37.5%), and medical supervision [observed medication] (38.6%) were less common; medication was frequently referral-based. Weekend availability was reported by 60.2% and parental/guardian consent requirements by 28.4%. Routine data readiness was higher in Lusaka than Shibuyunji (93.2% vs 66.7%; OR 6.8, 95% CI 1.7–27.7; p = 0.011). Forty-seven facilities were linked to ART sites representing 11,194 AYPLHIV: burden-weighted coverage was 40.7% for weekend services, 47.2% for referral medication, and 23.0% for a composite package (weekend availability + on-site counseling + referral medication). AYPLHIV burden is concentrated in Lusaka, but key access features (weekend options, privacy) and biomedical support beyond counseling often depend on referral pathways and are not consistently aligned with high-burden sites. Strengthening youth-friendly service organization, referral functionality, and routine monitoring at high-burden sites may improve equitable access to mental health and psychosocial support.

## Introduction

Adolescents and young people living with HIV (AYPLHIV) remain a priority population for HIV programs because they face ongoing risks to health and well-being, including challenges with long-term antiretroviral therapy (ART) adherence and retention. Globally, adolescents and young people represent a substantial share of the HIV epidemic and treatment need, with a large proportion of the burden concentrated in sub‑Saharan Africa [1,2].

Mental health conditions are common among AYPLHIV. In recent meta-analyses, approximately one-quarter of young people living with HIV experienced depression and about one in six experienced anxiety, with wide variation across settings and measurement tools [3,4]. These conditions may emerge or worsen in the context of HIV-related stigma, disclosure concerns, bereavement, developmental transitions, and the cumulative stress of living with a chronic condition.

Poor mental health is associated with adverse HIV outcomes, including suboptimal ART adherence and engagement in care, and can undermine the effectiveness of HIV service delivery approaches that rely on sustained self-management [3,5,6]. Psychosocial interventions (including counseling and peer support) have been shown to improve engagement and selected health and behavioral outcomes among AYPLHIV [6].

However, access to mental health care remains limited in many low- and middle-income settings due to underinvestment and severe workforce constraints. For example, specialist mental health providers for children and adolescents are scarce across much of sub‑Saharan Africa [7,8]. As a result, many programs rely on task-sharing and stepped-care approaches that integrate mental health and psychosocial support into primary care and HIV services.

Global guidance emphasizes integration of psychosocial interventions and support within HIV services for adolescents and young adults, including peer support, counseling, and clear referral pathways for more complex needs [5,9,10]. WHO’s mhGAP Intervention Guide provides an evidence-based framework for delivering mental health care in non-specialist settings through task sharing, which is particularly relevant in settings with limited specialist availability [11]. Standards for adolescent-friendly health services emphasize confidentiality, privacy, and service organization that aligns with adolescents’ schedules and reduce structural barriers to access [12].

In Zambia, health services are organized through a decentralized Ministry of Health structure, with national, provincial, district and facility levels responsible for planning, implementation and supervision of services. HIV care is delivered mainly through ART clinics located in public, faith-based and some private or partner-supported facilities, while mental health care is generally integrated into general outpatient, primary care and district-level services, with referral to higher-level or specialist services for more complex needs. Adolescents and young people may, therefore, encounter mental health support within HIV care, general primary care or referral pathways rather than through a separate adolescent mental health system. This organization is important for interpreting the mapping findings, because the presence of a service at a facility does not necessarily mean that all modalities, youth-friendly features, or referral pathways are available on site [13,14].

To support routine program planning and service strengthening, programs require granular information on what mental health and psychosocial support services are available for AYPLHIV, where services are delivered, when services are offered (including weekend access), who is eligible without barriers (age, sex, consent requirements), and how service availability aligns with the geographic distribution of AYPLHIV burden. We, therefore, mapped mental health and psychosocial service availability in Lusaka and Shibuyunji districts of Zambia and quantified alignment between service availability indicators and AYPLHIV burden using the programmatic treatment current (TX_CURR) indicator, a routine HIV program count of people currently receiving ART at the end of a reporting period [15,16].

This mapping analysis is part of a broader implementation research agenda to strengthen AYPLHIV access to mental health services in Zambia, including future work on mobile-enabled service navigation. The present manuscript does not evaluate or develop a mobile application. Instead, it provides foundational mapping data on service locations, hours, eligibility and consent requirements, ownership, and on-site versus referral pathways that could inform a future mobile service directory or referral-navigation tool [17–20].

## Materials and methods

### Ethics statement

Ethical approval for this study was obtained from the University of Zambia Biomedical Research Ethics Committee (UNZABREC), Reference No. 5864–2024, and from the National Health Research Authority (NHRA), Reference No. NHRA-1679/04/11/2024. In addition, clearance was obtained from the Ministry of Health at national, provincial, district and facility levels prior to data collection. Facility representatives who provided mapping information gave written informed consent before participation. They were informed about the voluntary nature of participation, their right to withdraw at any time, and measures taken to maintain confidentiality. No personal identifiers were collected for this facility-level analysis.

### Study design and setting

This analysis combined (1) a cross-sectional facility mapping of mental health and psychosocial support services relevant to AYPLHIV and (2) a secondary analysis of routine HIV program data to quantify AYPLHIV burden (TX_CURR). The work focused on Lusaka and Shibuyunji districts in Zambia.

Lusaka and Shibuyunji were selected purposively to capture contrasting service-delivery contexts within Zambia. Lusaka is predominantly urban and has the highest AYPLHIV ART program burden among the two study districts, while Shibuyunji is a more rural district with fewer service points and greater expected distance-related challenges. The two-district design was intended to support program planning by contrasting a high-volume urban setting with a lower-volume rural setting, to produce nationally representative estimates.

This manuscript reports the facility mapping and burden-alignment component, which serves as a foundational step for subsequent phases of the broader study focused on designing and testing a mobile application to facilitate linkage to youth-responsive mental health and psychosocial support services.

### Data sources and measures

#### Facility mapping survey.

A structured facility questionnaire was administered between 10 and 21 February 2025 to a roster of mental health and psychosocial support service providers in the two districts. The mapping captured facility identifiers, ownership type (government, private, other [Church owned]), geolocation, and service availability by modality and delivery pathway (on-site versus referral). Core service modalities included individual psychotherapy/counseling, group therapy, medication availability, and medical supervision (observed medication). The questionnaire also captured operating days (Monday–Sunday), eligibility constraints (sex eligibility, age bands served), consent requirements (e.g., parental/guardian consent for minors), availability of private space for counseling, and routine data readiness (whether services were recorded in routine paper-based or electronic systems, or reported through a mother/referral facility).

The structured facility questionnaire was developed specifically for this mapping exercise; it was not a direct adaptation of a single validated tool. Item domains were selected from the study objectives and aligned with programmatic domains emphasized in WHO and UNAIDS guidance on adolescent-friendly services and psychosocial support within HIV services, including service availability, referral pathways, confidentiality/privacy, operating hours, eligibility and routine documentation [5,10,12]. The draft tool was reviewed by the study team and local program stakeholders for contextual relevance before field use.

#### AYPLHIV burden (TX_CURR).

We extracted site-level TX_CURR data for ART sites in Lusaka and Shibuyunji for September 2024, which was accessed and retrieved on August 30^th^ 2025 for analysis. TX_CURR is a “snapshot” indicator defined as the number of persons currently receiving ART at the end of the reporting period [15,16]. For this manuscript, AYPLHIV burden was defined as the sum of TX_CURR for ages 15–19 and 20–24 years (15–24 total) with additional analyses reported for older youth (20–24 years) at the end of September 2024.

#### Data linkage between mapping and TX_CURR datasets.

Facility mapping records were linked to ART site identifiers in the routine program dataset using the aligned facility name and district. Facility names were standardized by converting to uppercase, trimming leading/trailing whitespace, collapsing multiple spaces, and removing punctuation to create a deterministic matching key. We then performed exact matching on standardized name and district. When multiple mapping records matched to the same ART site, we retained a single mapping record per ART site for burden-weighted analyses to avoid double counting of TX_CURR. The retained record was the first record in the cleaned dataset after deduplication, which reflected the ordering created during the data-cleaning process and should not be interpreted as random selection. This rule may introduce bias if duplicate records differed in their reported availability indicators; however, it was preferred to averaging or duplicating records because TX_CURR is reported once per ART site and duplicate inclusion would overstate the denominator contribution of those sites.

Facilities that could not be linked to ART site identifiers were retained in descriptive mapping analyses but excluded from burden-weighted alignment analyses. Consequently, burden-weighted results should be interpreted as applying to the linked ART-site subset only. If unlinked facilities differed systematically from linked sites (for example, if they were non-ART community, private or referral service points), the burden-weighted estimates may not represent the full mapped mental health service landscape.

All service-availability variables were based on facility self-report at the time of mapping. The survey did not independently verify staffing rosters, service registers, medication stock, referral completion, quality of care or actual service utilization. Facility self-report could therefore introduce information bias if respondents over-reported or under-reported services or access features.

### Statistical analysis

We summarized facility-level characteristics and service availability using counts and percentages overall and by district. Between-district comparisons of facility-level proportions used Pearson chi-square tests, with Fisher’s exact tests for 2 × 2 tables where expected cell counts were <5. For 2 × 2 comparisons, we additionally report unadjusted odds ratios (ORs) with Wald 95% confidence intervals. For the linked subset, we computed burden-weighted coverage as the proportion of AYPLHIV TX_CURR served at linked ART sites where the linked facility reported each availability indicator (numerator: TX_CURR among sites with the indicator; denominator: TX_CURR among all linked sites). Because TX_CURR reflects routine program counts rather than a sample, burden-weighted coverage estimates are presented descriptively without formal hypothesis testing. Statistical significance for facility-level comparisons was assessed at α = 0.05 (two-sided). Analyses were conducted in R (version 4.5.1) using RStudio (Posit) version 2025.05.1 + 513.

### Definitions and derived indicators

**Fig 1:**
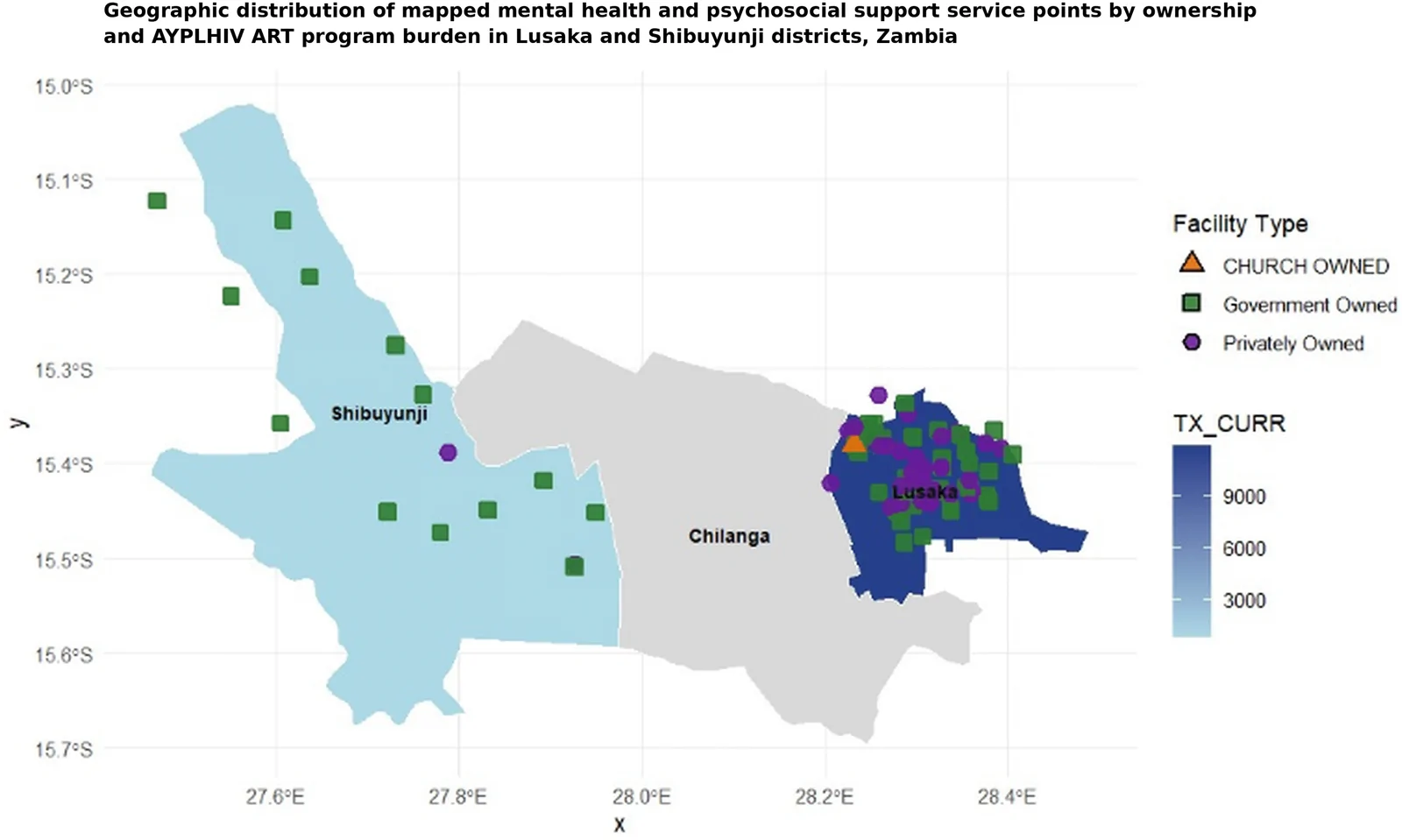
Facility landscape and distribution of mapped service points. Geographic distribution of mapped facilities providing mental health or psychosocial support services, symbolized by ownership type. Facility-level AYPLHIV burden at linked ART sites (TX_CURR, ages 15-24) is displayed using proportional circles to show where high-burden ART sites are located, rather than shading the whole district uniformly.

**Fig 2:**
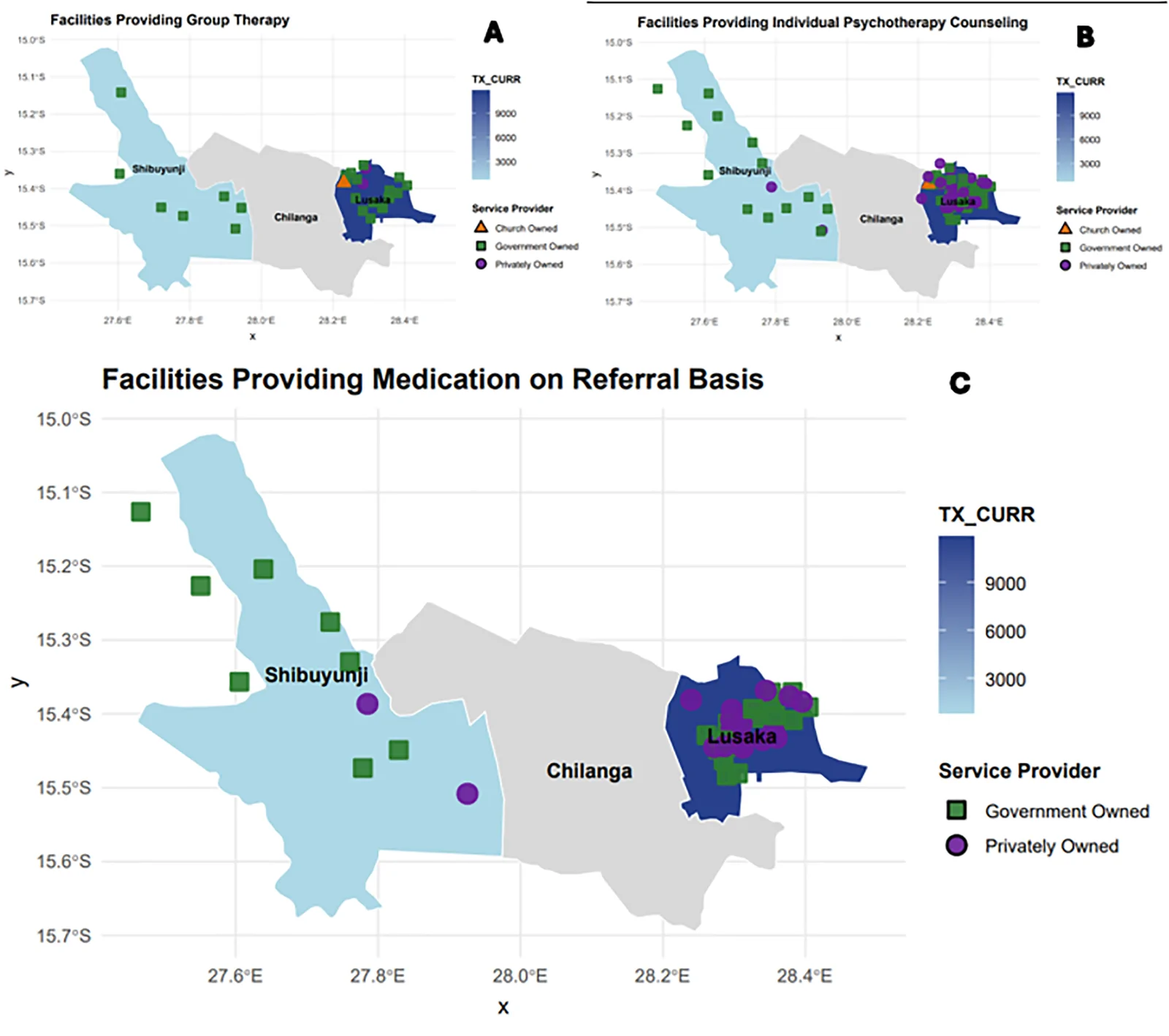
Availability by service modality and referral pathway. Maps of (A) facilities providing group therapy, (B) facilities providing individual psychotherapy/counseling, and (C) facilities providing medication on a referral basis. Facility points are symbolized by ownership type and linked ART-site TX_CURR is shown with proportional circles where applicable.

**Fig 3:**
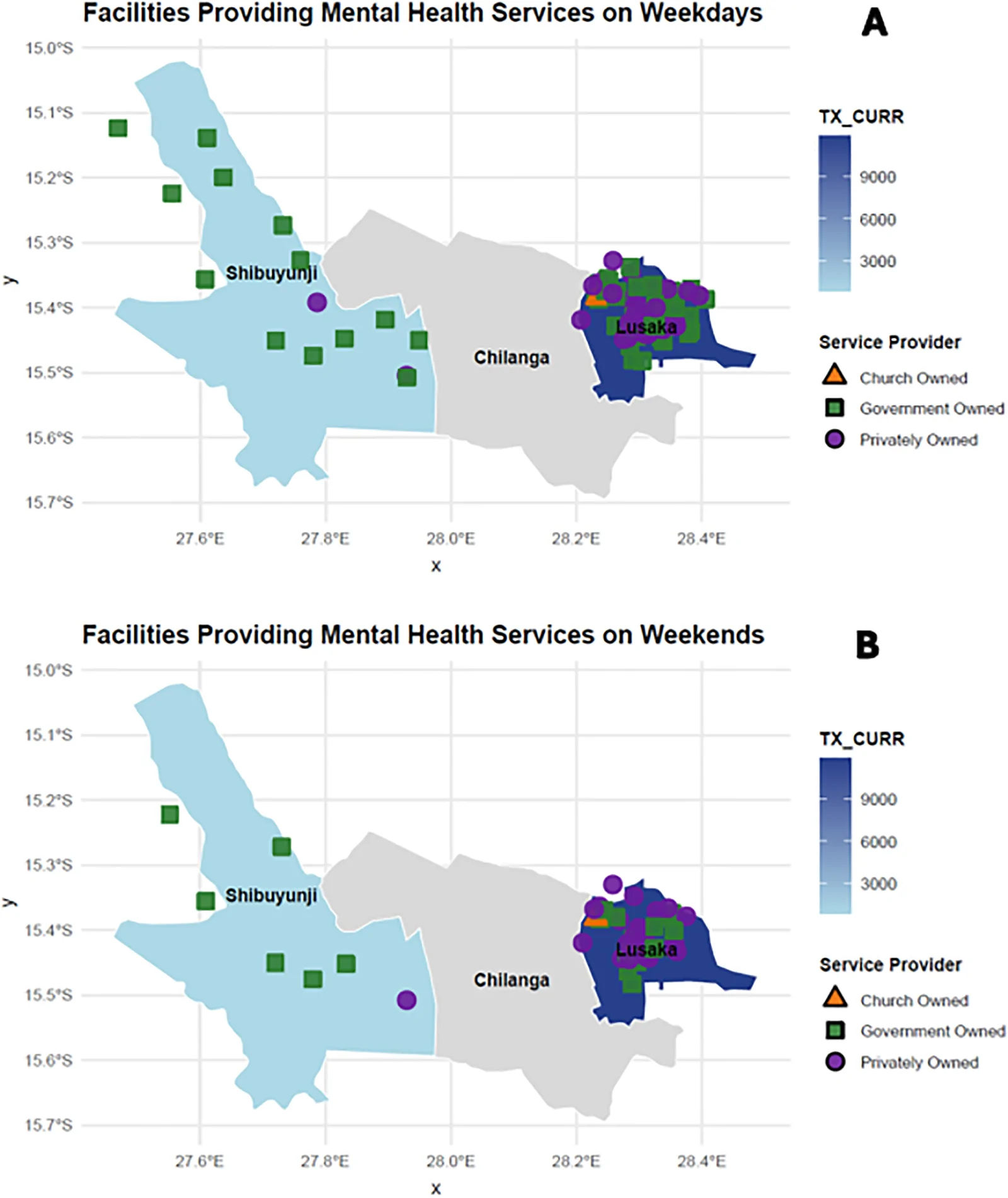
Availability by operating days. Maps of facilities providing mental health or psychosocial support services on (A) weekdays (Monday-Friday) and (B) weekends (Saturday and/or Sunday), with linked ART-site TX_CURR shown as proportional circles where applicable.

**Fig 4:**
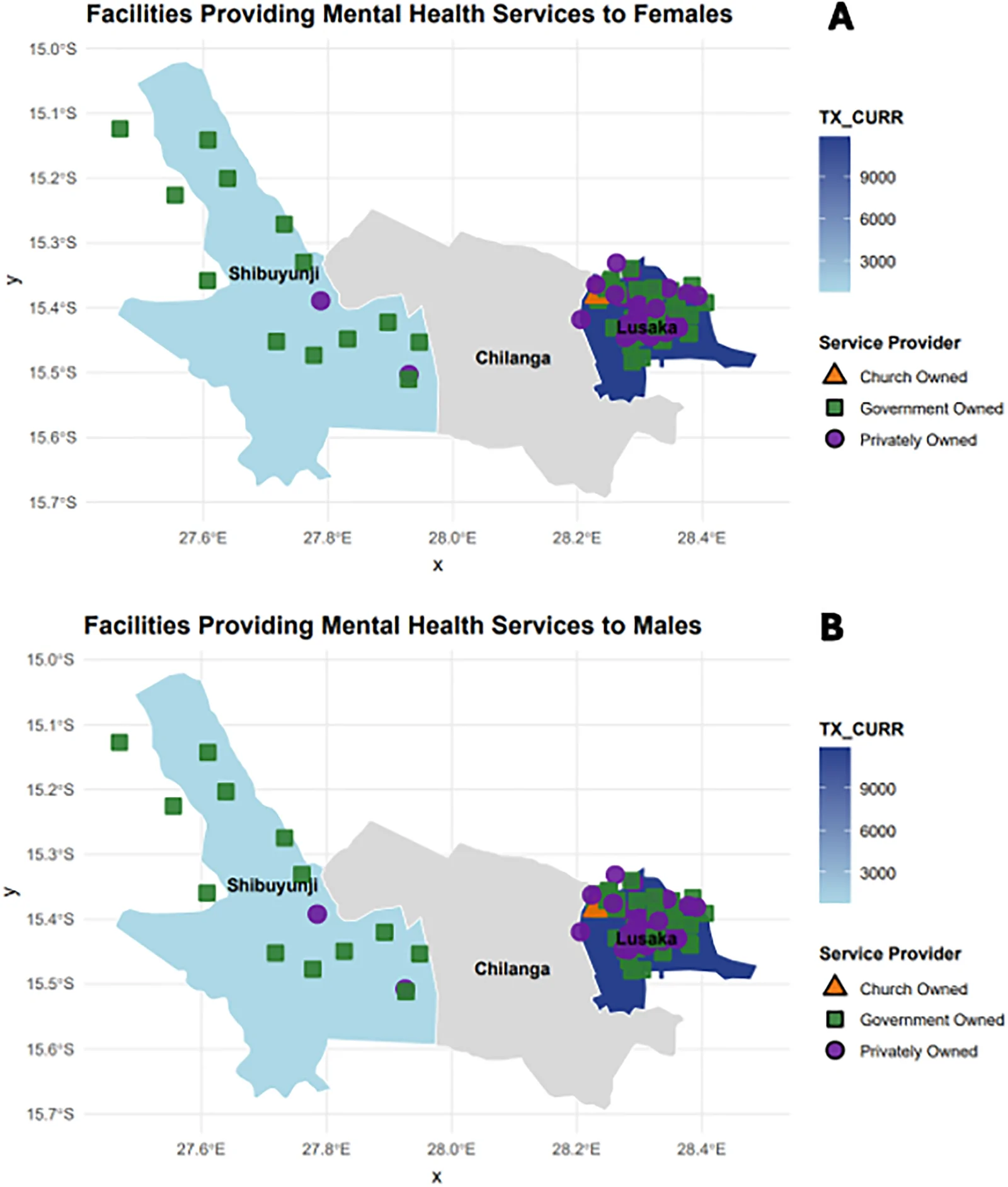
Service eligibility by sex. Maps of facilities reporting mental health or psychosocial support service eligibility for (A) females and (B) males, with facility ownership and linked ART-site TX_CURR shown where applicable.

**Fig 5:**
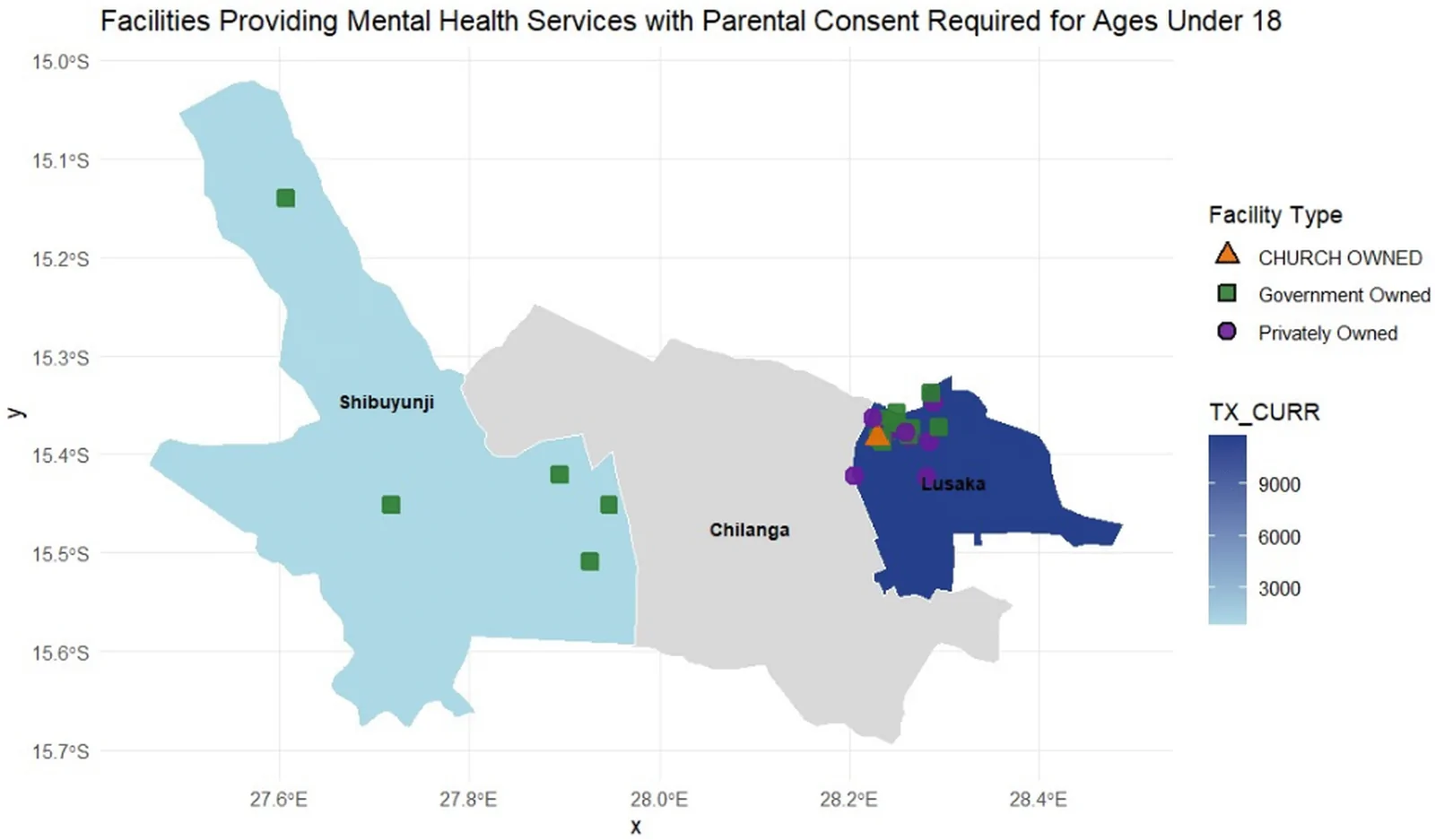
Parental/guardian consent requirements. Geographic distribution of mapped facilities reporting parental/guardian consent requirements for AYPLHIV under 18 years, with facility ownership and linked ART-site TX_CURR shown where applicable.

Key derived indicators are defined briefly below; full operational definitions and coding rules used for Tables 2, 3 and Figs 1–5 are provided in S1 Text.

- Availability indicator: facility-reported Yes/No availability of a service modality or access feature (e.g., weekend opening, on-site counseling, medication via referral).
- Linked subset: mapped facilities matched to routine program ART site identifiers with TX_CURR data; analyses are deduplicated to unique ART sites to avoid double counting.
- Burden-weighted coverage: proportion of AYPLHIV burden (TX_CURR) served at linked ART sites where the linked facility reports the indicator (numerator: TX_CURR among sites with the indicator; denominator: TX_CURR among all linked sites).
- Coverage gap: 1 minus burden-weighted coverage, representing the share of linked AYPLHIV burden served at sites without the indicator.
- Composite availability package: weekend availability (Saturday and/or Sunday) AND on-site individual psychotherapy/counseling AND medication available through referral.

### Mapping and visualization

Facility locations were mapped using geocoordinates captured during the facility survey and administrative district boundaries. Facilities were symbolized by ownership and availability indicators (e.g., counseling on-site, referral-based medication, weekend services). For maps that include AYPLHIV burden, facility-level TX_CURR at linked ART sites was shown using proportional circles to avoid implying uniform burden across an entire district. References to facility density or dispersion are descriptive, based on the mapped point distribution and district context, and were not derived from formal spatial-dispersion statistics. Map figures were organized to support interpretation along the dimensions of what services are available, where services are delivered, and when services are accessible.

## Results

This results section describes mental health and psychosocial support services available to AYPLHIV in Lusaka and Shibuyunji districts, Zambia. It emphasizes AYPLHIV ART program burden (TX_CURR, ages 15–24) as a programmatic marker of need; what services are available; where and when services are provided; who can access services without age, sex or consent-related barriers; and how ownership and routine documentation may shape access. Detailed values are presented in the tables; the narrative highlights the main patterns relevant for program planning.

### AYPLHIV burden (TX_CURR 15–24): Need for mental health services

Across 87 ART sites in Lusaka and Shibuyunji districts, there were 16,550 AYPLHIV aged 15–24 currently on ART (TX_CURR, as of September 2024). The majority of AYPLHIV burden was in Lusaka (16,167; 97.7%) compared with Shibuyunji (383; 2.3%). Overall, females accounted for 11,564 (69.9%) of AYPLHIV aged 15–24 on ART, and males accounted for 4,986 (30.1%). Older youth aged 20–24 accounted for 11,313 (68.4%) and adolescents aged 15–19 accounted for 5,237 (31.6%).

AYPLHIV burden was concentrated in a subset of high-volume sites (median TX_CURR 15–24 per site: 82; IQR: 38–209; range: 1–1,826). The top 10 ART sites accounted for 48.5% of district-wide AYPLHIV burden (15–24) (top 5 sites: 31.4%).

Table 1 summarizes TX_CURR by age group and sex for the two districts.

**Table 1:** AYPLHIV ART program burden (TX_CURR, ages 15–24) by age group and sex, September 2024. Values are counts of AYPLHIV on ART (TX_CURR) as at the end of September 2024. Age groups reflect standard MOH reporting in five-year age intervals for adolescents and young people (15–19 and 20–24).

Age group	Lusaka Female	Lusaka Male	Lusaka Total	Shibuyunji Female	Shibuyunji Male	Shibuyunji Total	Total
15-19 yrs	2,985 (57.0%)	2,099 (40.1%)	5,084 (97.1%)	100 (1.9%)	53 (1.0%)	153 (2.9%)	5,237
20-24 yrs	8,300 (73.4%)	2,783 (24.6%)	11,083 (98.0%)	179 (1.6%)	51 (0.5%)	230 (2.0%)	11,313
Total	11,285 (68.2%)	4,882 (29.5%)	16,167 (97.7%)	279 (1.7%)	104 (0.6%)	383 (2.3%)	16,550

Chi-square test comparing district-by-age-and-sex distribution (ages 15–24): p < 0.001 (descriptive comparison of program totals).

**Source**: Computed from program data.

#### Where services are provided: Mapped service landscape and ownership.

A total of 88 facilities reporting provision of any mental health or psychosocial support service were mapped across the two districts (Lusaka: 73; Shibuyunji: 15). The mapped points showed a larger number of facilities clustered in urban Lusaka and fewer facilities spread across Shibuyunji. This description reflects the observed mapped point pattern and service counts rather than a formal spatial dispersion test. Ownership differed by district: government-owned facilities represented a larger share of mapped facilities in Shibuyunji, whereas privately owned facilities were more prominent in Lusaka (Table 2).

**Table 2:** Availability of mental health and psychosocial support services for AYPLHIV at mapped facilities, Lusaka and Shibuyunji. Values are n (%), unless otherwise noted. P-values compare Lusaka vs Shibuyunji (Pearson chi-square or Fisher’s exact tests as appropriate). “On-site” indicates the service was reported as available at the facility itself; “through referral” indicates the facility reported referring clients elsewhere to access the service. “Routine data readiness” indicates whether the facility reported routinely recording mental health services in a paper-based or electronic system (or reporting through a mother/referral facility).

Indicator	Lusaka (N = 73) n/N (%)	Shibuyunji (N = 15) n/N (%)	Total (N = 88) n/N (%)	p-value
Total mapped facilities (N)	73	15	88	
Ownership				
Government Owned	38/73 (52.1%)	13/15 (86.7%)	51/88 (58.0%)	0.046
Privately Owned	34/73 (46.6%)	2/15 (13.3%)	36/88 (40.9%)	0.021
Other (Church owned)	1/73 (1.4%)	0/15 (0.0%)	1/88 (1.1%)	1.000
Total	**73**	**15**	**88**	
Eligibility & consent				
No age restrictions reported (No age requirements)	68/73 (93.2%)	15/15 (100.0%)	83/88 (94.3%)	0.583
Parental/guardian consent required for any age band (derived)	20/73 (27.4%)	5/15 (33.3%)	25/88 (28.4%)	0.755
Consent required for ages 10–14	19/73 (26.0%)	5/15 (33.3%)	24/88 (27.3%)	0.542
Consent required for ages 15–17	3/73 (4.1%)	2/15 (13.3%)	5/88 (5.7%)	0.200
Consent required for ages 20–24	1/73 (1.4%)	0/15 (0.0%)	1/88 (1.1%)	1.000
Operating days				
Weekdays (Mon–Fri)	73/73 (100.0%)	15/15 (100.0%)	88/88 (100.0%)	1.000
Weekends (Sat and/or Sun)	46/73 (63.0%)	7/15 (46.7%)	53/88 (60.2%)	0.374
Saturday	44/73 (60.3%)	7/15 (46.7%)	51/88 (58.0%)	0.493
Sunday	29/73 (39.7%)	5/15 (33.3%)	34/88 (38.6%)	0.863
On-site service availability				
Individual psychotherapy/counseling	71/73 (97.3%)	15/15 (100.0%)	86/88 (97.7%)	1.000
Group therapy	21/73 (28.8%)	7/15 (46.7%)	28/88 (31.8%)	0.225
Medication	27/73 (37.0%)	6/15 (40.0%)	33/88 (37.5%)	1.000
Medical supervision	29/73 (39.7%)	5/15 (33.3%)	34/88 (38.6%)	0.863
Recreational therapies	10/73 (13.7%)	4/15 (26.7%)	14/88 (15.9%)	0.247
Private room available	39/73 (53.4%)	9/15 (60.0%)	48/88 (54.5%)	0.856
Referral service availability				
Individual psychotherapy /counseling	16/73 (21.9%)	6/15 (40.0%)	22/88 (25.0%)	0.19
Medication	35/73 (47.9%)	10/15 (66.7%)	45/88 (51.1%)	0.299
Medical supervision	15/73 (20.5%)	5/15 (33.3%)	20/88 (22.7%)	0.316
Routine data readiness				
Any mental health service	68/73 (93.2%)	10/15 (66.7%)	78/88 (88.6%)	0.011
Counseling	67/73 (91.8%)	10/15 (66.7%)	77/88 (87.5%)	0.019
Medication	22/73 (30.1%)	3/15 (20.0%)	25/88 (28.4%)	0.541
Medical supervision	17/73 (23.3%)	3/15 (20.0%)	20/88 (22.7%)	1.000

**Source**: Computed.

Fig 1 shows the spatial distribution of mapped facilities by ownership type, with facility-level TX_CURR displayed at linked ART sites using proportional symbols to contextualize service locations against AYPLHIV care burden.

#### What services are available to AYPLHIV (and challenges, including referral dependence and information gaps).

Table 2 summarizes facility-reported service availability and key access characteristics. Indicators are not mutually exclusive; facilities may report multiple services and access features. The main pattern was a counseling-forward service landscape: individual psychotherapy/counseling was widely available on site, while group therapy, medication, medical supervision and private space were less consistently reported.

Referral pathways were common, and medication was frequently cited as referral-based (51.1%). This pattern indicates a counseling-forward service package with substantial reliance on referrals for pharmacologic management and specialized support, which may introduce additional steps for AYPLHIV (e.g., transport and appointment delays) compared with on-site services.

Information on service delivery was not uniformly available across modalities. Overall, 88.6% of facilities reported routinely collecting some mental health service data, and routine data readiness was higher in Lusaka than Shibuyunji (93.2% vs 66.7%; OR 6.8, 95% CI 1.7–27.7; p = 0.011). Routine data capture was highest for individual counseling (87.5%) and lower for medication (28.4%) and medical supervision (22.7%), suggesting gaps in routine documentation for key service components.

The geographic distribution of selected service modalities and referral pathways is shown in Fig 2.

#### When services are provided: Operating days and potential access gaps.

Most facilities reported weekday availability, whereas weekend availability was more constrained (Fig 3). This pattern may be important for AYPLHIV who have weekday constraints related to school, work, caregiving or transport, but the mapping data do not directly measure whether weekend opening changes service use.

#### Who can access services: Eligibility by sex and consent requirements.

Eligibility restrictions by sex were uncommon; nearly all facilities reported services were available to both females and males (Fig 4). Most facilities reported no age restriction, but a minority reported parental/guardian consent requirements, especially for younger adolescents (Fig 5). These findings identify potential administrative and confidentiality-related constraints, but do not measure actual help-seeking or service completion.

The geographic distribution of facilities reporting parental/guardian consent requirements is shown in Fig 5.

### Alignment of service availability with AYPLHIV burden (TX_CURR): Coverage and gaps

To quantify alignment of service availability with AYPLHIV program burden, we linked mapped facilities to MOH reporting system site identifiers and September 2024 TX_CURR. Overall, 65/88 (73.9%) mapped facilities matched to an ART site in the MOH reporting system, yielding 47 unique linked ART sites. The linked subset represented 11,194 AYPLHIV aged 15–24 in care. Results below are limited to this linked ART-site subset and should not be interpreted as representing all mapped service points.

Among linked facilities, 38/47 (80.9%) were government-owned and served 9,645/11,194 (86.2%) of linked AYPLHIV burden (15–24). Privately owned facilities accounted for 8/47 (17.0%) of linked facilities and served 1,497/11,194 (13.4%) of linked AYPLHIV burden (15–24).

Weekend service availability (open Saturday and/or Sunday) covered 4,554/11,194 (40.7%) of linked AYPLHIV burden (15–24), implying that 6,640/11,194 (59.3%) of burden was served at facilities without weekend availability. Medication access via referral covered 5,287/11,194 (47.2%) of linked burden (gap: 5,907/11,194; 52.8%). The composite availability package (weekend availability + on-site counseling + referral medication) covered 2,570/11,194 (23.0%) linked burden, leaving a composite coverage gap of 8,624/11,194 (77.0%).

For older youth aged 20–24 within the linked subset (n = 7,811), weekend availability covered 3,231/7,811 (41.4%) (gap: 58.6%), referral-based medication covered 3,722/7,811 (47.7%) (gap: 52.3%), and the composite package covered 1,848/7,811 (23.7%) (gap: 76.3%) (Table 3).

**Table 3:** Alignment of key availability indicators with AYPLHIV burden (TX_CURR) among linked facilities. Panel A reports facility-level prevalence of each availability indicator among linked mapped facilities (i.e., facilities reporting “Yes” to the indicator in the mapping survey). Panels B and C report burden-weighted coverage, defined as the proportion of AYPLHIV burden (TX_CURR) served at linked ART sites where the linked facility reported the indicator. For each cell, the numerator is TX_CURR at sites with the indicator and the denominator is TX_CURR across all linked sites. Burden-weighted values are presented descriptively without formal hypothesis testing. The composite availability package is defined as weekend availability (Saturday and/or Sunday) AND on-site individual psychotherapy/counseling AND medication available through referral. Panel A. Facility-level prevalence of availability indicators among linked mapped facilities (n = 47).

Indicator	Lusaka	Shibuyunji	Total	p-value
Panel A. Facility-level service availability prevalence among linked facilities (n/N)				
Denominator (N linked facilities)	36	11	47	
Weekend services (Sat and/or Sun)	19/36 (52.8%)	6/11 (54.5%)	25/47 (53.2%)	1.000
Individual counseling on-site	35/36 (97.2%)	11/11 (100.0%)	46/47 (97.9%)	1.000
Medication available via referral	18/36 (50.0%)	8/11 (72.7%)	26/47 (55.3%)	0.300
Composite (weekend + counseling + referral medication)	11/36 (30.6%)	6/11 (54.5%)	17/47 (36.2%)	0.275
Panel B. Burden-weighted coverage (TX_CURR 15–24) among linked facilities				
Denominator (TX_CURR 15–24, linked burden)	10,811	383	11,194	
Weekend services (Sat and/or Sun)	4,379/10,811 (40.5%)	175/383 (45.7%)	4,554/11,194 (40.7%)	—
Individual counseling on-site	10,775/10,811 (99.7%)	383/383 (100.0%)	11,158/11,194 (99.7%)	—
Medication available via referral	5,027/10,811 (46.5%)	260/383 (67.9%)	5,287/11,194 (47.2%)	—
Composite (weekend + counseling + referral medication)	2,395/10,811 (22.2%)	175/383 (45.7%)	2,570/11,194 (23.0%)	—
Panel C. Burden-weighted coverage (TX_CURR 20–24) among linked facilities				
Denominator (TX_CURR 20–24, linked burden)	7,581	230	7,811	
Weekend services (Sat and/or Sun)	3,129/7,581 (41.3%)	102/230 (44.3%)	3,231/7,811 (41.4%)	—
Individual counseling on-site	7,550/7,581 (99.6%)	230/230 (100.0%)	7,780/7,811 (99.6%)	—
Medication available via referral	3,571/7,581 (47.1%)	151/230 (65.7%)	3,722/7,811 (47.7%)	—
Composite (weekend + counseling + referral medication)	1,746/7,581 (23.0%)	102/230 (44.3%)	1,848/7,811 (23.7%)	—
Panel D. Ownership distribution among linked facilities and linked burden (TX_CURR 15–24)				
Panel D1. Facilities (n/N) by ownership				
Denominator (N linked facilities)	36	11	47	
Government Owned	29/36 (80.6%)	9/11 (81.8%)	38/47 (80.9%)	—
Privately Owned	6/36 (16.7%)	2/11 (18.2%)	8/47 (17.0%)	—
Other (Church owned)	1/36 (2.8%)	0/11 (0.0%)	1/47 (2.1%)	—
Panel D1 total	**36**	**11**	**47**	**—**
Panel D2. Linked AYPLHIV burden served (TX_CURR 15–24) by ownership				
Denominator (TX_CURR 15–24, linked burden)	10,811	383	11,194	
Government Owned	9,315/10,811 (86.2%)	330/383 (86.2%)	9,645/11,194 (86.2%)	—
Privately Owned	1,444/10,811 (13.4%)	53/383 (13.8%)	1,497/11,194 (13.4%)	—
Other (Church owned)	52/10,811 (0.5%)	0/383 (0.0%)	52/11,194 (0.5%)	—
Panel D2 total	**10,811**	**383**	**11,194**	**—**

**Source**: Computed.

## Discussion

### Principal findings

This facility mapping and burden-alignment analysis provides a programmatically actionable description of mental health and psychosocial support services available to AYPLHIV in Lusaka and Shibuyunji districts. We found that AYPLHIV burden (TX_CURR 15–24) was highly concentrated in Lusaka and clustered within a subset of high-volume ART sites, underscoring the importance of prioritizing service strengthening at facilities serving the largest volumes of adolescents and young adults on treatment.

Across mapped facilities, individual counseling was widely reported and commonly delivered on-site, while other modalities, including group therapy, medical supervision, and medication availability, were less consistently available and, when available, were frequently offered via referral pathways. This pattern suggests that, for many AYPLHIV, the effective “package” of mental health services may depend on the functionality of referral networks rather than on-site integration.

Operating days and accessibility were key potential access constraints. Weekend availability was not universal, particularly in Shibuyunji, even though adolescents and young adults may face weekday barriers related to school, work, and transport. In addition, a non-trivial proportion of facilities reported parental/guardian consent requirements for younger adolescents, raising the possibility of delayed or foregone care among those who cannot or prefer not to seek caregiver involvement.

Finally, ownership patterns differ by district and have equity implications. Most mapped and linked AYPLHIV burden was served at government-owned facilities, which implies that improvements in public sector readiness and integration could reach the largest share of AYPLHIV in care. At the same time, privately owned facilities represented a substantial portion of the mapped service landscape in Lusaka, which may influence access and referral dynamics (including potential financial barriers), even though patient fees were not assessed in the mapping survey.

### Programmatic implications

WHO guidance recommends integrating psychosocial interventions and support into HIV services for adolescents and young adults, including counseling, peer support, and clear referral pathways for higher-intensity or specialized needs [5,9,10]. The observed reliance on referral pathways for medication and other specialized services highlights the need to strengthen referral systems (documentation, feedback loops, and continuity of care) and to clarify “what is available where” for frontline HIV providers and clients.

The findings are consistent with emerging evidence from other sub-Saharan African settings that emphasizes the need for adolescent- and youth-responsive mental health promotion, community-based psychosocial support and clearer service-navigation pathways. In Mozambique, a community-based psychodrama intervention involving adolescents and young adults, including those living with HIV, improved mental health knowledge and emotional awareness and was feasible and acceptable in a resource-limited context [21]. A separate Mozambique study using the COM-B framework found that barriers to mental health service access among young people living with HIV and providers included community stigma, staff shortages, limited community services, distance from health centres and limited knowledge of available services [22]. Our mapping findings provide complementary supply-side evidence by showing where services are available, which modalities depend on referral and where routine documentation gaps remain.

Given persistent workforce constraints in many settings, task-sharing frameworks such as WHO’s mhGAP can support stepped-care delivery of mental health services in non-specialist facilities, with escalation pathways for complex cases [8,11]. In these districts, expanding the competency of existing cadres (e.g., lay counselors, nurses, clinical officers) to deliver evidence-based psychosocial interventions may be a feasible pathway to increase effective service availability where psychiatric specialists are limited.

Service organizations should also consider adolescent-friendly access features. Global standards for quality adolescent health services emphasize confidentiality and privacy, as well as service organization that reduces structural barriers and aligns with adolescents’ schedules [12]. The observed gaps in weekend service availability and private counseling space suggest specific, operationally tractable targets for improvement at high-burden sites (e.g., weekend clinic sessions for adolescents and young adults; dedicated private rooms or time slots for counseling).

### Equity and access considerations

Consent requirements for younger adolescents represent a potential barrier to timely access, particularly for those experiencing stigma, family conflict, or concerns about confidentiality. While consent frameworks for minors may reflect legal and policy expectations for health services, programs can still implement adolescent-friendly approaches, such as clear communication of consent processes, confidential counseling where permitted, and supportive caregiver engagement strategies, to reduce deterrents to care [12,23].

### Implications for mobile application–enabled access and linkage

The mapping results provide practical information that could inform future mobile-enabled service navigation, but the present study did not evaluate a mobile intervention. Potential future uses include a regularly updated service directory, display of facility operating days and eligibility requirements, referral navigation, appointment coordination and targeted client communication. These should be treated as candidate applications for future feasibility, acceptability and implementation research, not as interventions proven by this mapping study. Because service hours, staffing and referral pathways can change over time, any mobile-enabled directory would require governance arrangements for periodic updating and verification.

### Routine data readiness and monitoring

Although many facilities reported routinely documenting counseling services, routine data capture for other service modalities (including medication and group therapy) was less common. Routine data readiness was significantly higher in Lusaka than Shibuyunji (93.2% vs 66.7%; OR 6.8, 95% CI 1.7–27.7; p = 0.011), highlighting the need to strengthen registers, staff capacity, and reporting workflows in lower-resource settings. Strengthening routine monitoring for mental health and psychosocial support services, including consistent definitions, registers, and reporting flows, would support more precise planning, supervision, and evaluation. Alignment with standard program indicator definitions (including TX_CURR and relevant service delivery indicators) may facilitate integration into existing HIV monitoring systems [15,16].

### Strengths and limitations

A key strength of this work is the integration of facility-level mapping with routine program burden data to move beyond a purely descriptive service inventory and towards a quantified assessment of “services aligned with need”. The geospatial presentation further supports district-level planning by making geographic patterns of access constraints visible.

Limitations should be considered when interpreting the findings. First, service availability was measured by facility self-report at one point in time and was not independently verified through register review, observation, staffing data, stock records, referral completion or client utilization data; information bias is therefore possible. Second, not all mapped facilities could be linked to routine program site identifiers, and duplicate matching was resolved using the first cleaned mapping record; these decisions may bias burden-weighted analyses if linked, unlinked or duplicate records differed systematically. Third, TX_CURR reflects ART program burden among AYPLHIV and is an imperfect proxy for mental health need; it does not measure symptoms, diagnoses, perceived need, service utilization or outcomes. Fourth, the mapping did not systematically measure fees, transport time, waiting time, provider competency, or quality of care, all of which influence real-world access. Finally, statistical comparisons were exploratory and based on relatively small district-specific counts, especially in Shibuyunji; p-values and confidence intervals should therefore be interpreted as descriptive signals rather than confirmatory evidence.

### Next steps

Future work should incorporate measures of service utilization and quality, and, where feasible, link facility availability to client-level outcomes such as retention and viral suppression. Additional data on affordability and on adolescent/youth acceptability (including confidentiality and stigma concerns) would further strengthen the ability to interpret “availability” as true access. As a complementary strategy, the mapped service directory and availability indicators can be translated into a mobile application prototype to support service discovery, referral navigation, and appointment coordination; subsequent phases should evaluate acceptability, feasibility, and equity of access among AYPLHIV and providers.

## Conclusions

This facility mapping and burden-alignment analysis documents the mental health and psychosocial support service landscape relevant to AYPLHIV in Lusaka and Shibuyunji districts and identifies practical availability gaps across what is offered, where services are delivered, and when services are available. AYPLHIV ART burden was concentrated in Lusaka and within high-volume ART sites, while service availability beyond counseling often depended on referral pathways and weekend availability was not universal. Strengthening integrated psychosocial support within HIV services, improving referral functionality, expanding adolescent-friendly access features (including privacy and feasible service hours), and improving routine monitoring of mental health services may improve alignment of services with AYPLHIV program burden. These findings should be interpreted as service-availability and program-planning evidence rather than direct evidence of mental health need, service utilization or intervention effectiveness.

### Use of large language models

We used a large language model (ChatGPT, OpenAI) to assist with language editing and formatting of the manuscript; all authors reviewed and are responsible for the final content.

## Supporting information

S1 Data De-identified facility-level mapping dataset used to generate facility-level availability indicators, tables and map figures. (CSV)

S2 Data Site-level TX_CURR age/sex dataset for FY2024 Q4/end of September 2024 used for Table 1 and burden-weighted analyses. (CSV)

S3 Data Wide-format site-level TX_CURR totals for ages 15–19, 20–24 and combined 15–24. (CSV)

S4 Data District-level TX_CURR dataset used for map shading/context. (CSV)

S5 Data Administrative boundary GeoPackage used to create map figures. (GPKG)

S1 Text Supporting notes describing the datasets and coding conventions. (TXT)

S1 Code R script used to reproduce derived indicators, tables and summary analyses. (R)

S2 Code R script used to reproduce map and figure-related outputs. (R)
