# Supplementary material for: Mapping mental health and psychosocial support service availability for adolescents and young people living with HIV in Lusaka and Shibuyunji districts, Zambia: Alignment with ART program burden (September 2024)
Source: PLOS Ment Health. 2026 Aug 25;3(8):e0000610. doi: 10.1371/journal.pmen.0000610 (PMC13505908; doi:10.1371/journal.pmen.0000610)
Supplement: Supplementary file 1 [file pmen.0000610.s010.pdf]

Response to Reviewers

Manuscript ID: PMEN-D-26-00221

PLOS Mental Health

Dear Dr. Montague-Cardoso and Reviewers,

Note: Line numbers cited below refer to the clean revised manuscript file.

Thank you for the careful review of our manuscript. We appreciate the constructive feedback and have revised the manuscript to improve contextual clarity, methods transparency, interpretation of the results, figure interpretation, data availability and PLOS submission compliance. We provide below a point-by-point response. All changes are included in the clean revised manuscript and are highlighted in the marked-up version.

Journal requirements

Comment	Response	Manuscript change
Ethics Statement must be available in its entirety at the beginning of Methods under the subheading "Ethics Statement".	Addressed. We moved the full ethics statement to the beginning of the Methods section and used the required subheading.	Ethics Statement moved to the beginning of Methods under the required subheading; see lines 109-119.
Financial disclosure must be completed in full sentences, with initials of authors receiving funding/salary and funder role.	Addressed. We added a full Financial Disclosure statement. The statement names the Fogarty/NIH award, identifies CM as receiving doctoral training support, states that no other authors received salary/direct funding from the award for this work, and clarifies that the funder had no role in the research or publication process.	Financial Disclosure added in full sentences; see lines 511-516.
Please provide separate figure files in .tif or .eps format.	Noted. Figure captions and text have been updated. Separate figure files should be uploaded during resubmission following PLOS figure requirements and processed through the PLOS figure checking tool where possible.	Figure captions revised in the manuscript (Figs 1-5; see lines 269-273, 303-307, 313-316, 323-330), and separate high-resolution TIFF figure files are provided for upload.
Please confirm whether all raw data required to replicate the results are provided, or explain restrictions.	Addressed. We revised the Data Availability statement to specify that all data required to replicate the findings are provided as Supporting Information: facility-level mapping data, site-level TX_CURR and linked analytic data, and R analysis code.	Data Availability statement revised to identify the minimal datasets and code supplied as Supporting Information; see lines 517-523.

Reviewer #1

Comment	Response	Manuscript change
For readers unfamiliar with Zambia, briefly explain how health services are organized, particularly for adolescents and with regard to HIV and mental health.	Addressed. We added contextual information explaining Zambia's decentralized Ministry of Health structure; ART service delivery through public, faith-based, private and partner-supported sites; and the integration/referral organization of mental health services.	New Zambia health-system context paragraph added to Introduction; see lines 81-92.

Were bibliographical references used in developing the structured questionnaire? If so, cite them; if not, explain the process in more detail.	Addressed. We clarified that the questionnaire was not directly adopted from a single validated tool. It was developed from study objectives, programmatic domains in WHO/UNAIDS guidance, and local stakeholder review.	Questionnaire development process and guidance sources clarified under Facility mapping survey; see lines 149-156.
The statement that facilities were more densely distributed in Lusaka and more geographically dispersed in Shibuyunji is unclear; make assessment explicit.	Addressed. We revised the Methods and Results to clarify that density/dispersion refers to the descriptive mapped point pattern and service counts, not a formal spatial-dispersion statistic.	Dispersion wording revised to clarify this was a descriptive mapped point pattern, not a formal spatial dispersion test; see Methods lines 220-228 and Results lines 258-263.
Bibliography is limited; expand the discussion by comparing with sub-Saharan African experiences, including suggested DOIs.	Addressed. We added the two suggested publications from Mozambique and expanded the Discussion to compare our supply-side mapping findings with evidence on community-based mental health promotion and barriers/facilitators to mental health access among young people living with HIV.	Discussion expanded to compare with other sub-Saharan African evidence, including the two suggested Mozambique papers; see lines 403-415. New references are included as references 22-23; see lines 598-604.
Mention whether the matching criterion "first record in the cleaned dataset" could introduce bias or whether it is random.	Addressed. We clarified that the rule was not random and may introduce bias if duplicate records differed. We explained the rationale: TX_CURR is reported once per ART site, so retaining one record avoided double counting.	Matching rule clarified as non-random and potential bias noted; see linkage Methods lines 169-177 and limitations lines 468-471.

Reviewer #2

Comment	Response	Manuscript change
Explain why Lusaka and Shibuyunji were selected.	Addressed. We added that the districts were purposively selected to represent contrasting urban/rural service-delivery contexts, with Lusaka having the higher AYPLHIV ART program burden and Shibuyunji representing a more rural context with fewer service points.	District selection rationale added; see lines 125-131.
Define TX_CURR at first mention.	Addressed. TX_CURR is now defined in the Introduction as a routine HIV program count of people currently receiving ART at the end of a reporting period; the Methods definition is retained.	TX_CURR defined at first substantive mention and again in Methods; see lines 100-102 and 157-163.
Mention the future mobile application more cautiously because the paper does not evaluate or develop the app.	Addressed. We revised the Introduction and Discussion to state that the mapping provides foundational data that could inform a future mobile service directory or referral-navigation tool; it does not evaluate a mobile intervention.	Mobile application language revised to be cautious and foundational rather than evaluative; see lines 103-108 and the Discussion lines 437-446.
Explain rationale for retaining the first record when multiple facilities matched a single ART site.	Addressed. We expanded the linkage methods to explain that the rule avoided double counting TX_CURR and that the retained record was not random.	Rationale for first-record selection added, including that it avoids double counting TX_CURR and is not random; see lines 169-177.
Discuss whether facility self-report could have introduced measurement bias.	Addressed. We added this explicitly in the Methods and limitations.	Facility self-report measurement bias addressed in Methods and limitations; see lines 184-188 and 464-467.
Acknowledge in Methods that excluding unmatched facilities from linkage analyses could influence burden-weighted results.	Addressed. We added a paragraph after the linkage description stating that unlinked facilities were retained in descriptive mapping but excluded from burden-weighted analyses, limiting interpretation to the linked subset.	Methods now state that unlinked facilities were retained for descriptive mapping but excluded from burden-weighted analyses, and how this affects interpretation; see

		lines 178-183.
Some Results narrative is repetitive with tables; consider shortening.	Addressed. We shortened several Results paragraphs and clarified that the text highlights patterns while detailed values remain in tables.	Results narrative shortened and reframed to highlight main patterns while detailed values remain in tables; see lines 231-238 and revised Results subsections thereafter.
Avoid overinterpreting statistical significance given sample size and exploratory comparisons.	Addressed. We strengthened the limitations to state that statistical comparisons are exploratory, especially in Shibuyunji, and should be interpreted as descriptive signals rather than confirmatory evidence.	Statistical comparisons explicitly framed as exploratory in limitations; see lines 475-478.
The mobile application section is speculative; frame as future applications and acknowledge need for regular updating.	Addressed. We reframed this section as potential future uses informed by mapping, not proven functions. We added that a mobile directory would need governance for periodic updating and verification because services, hours and referral pathways can change.	Mobile application implications reframed as future feasible uses needing implementation research and periodic updating; see lines 437-446 and next steps lines 484-488.
Emphasize that TX_CURR is a proxy for HIV program burden, not mental health burden.	Addressed. We added this caution in the limitations and conclusion.	Limitations and conclusion now clarify that TX_CURR reflects HIV program burden and not mental health need, symptoms, utilization or outcomes; see lines 471-473 and 499-501.
Figures: consider whether five maps are necessary; several communicate similar spatial patterns.	Addressed. We reduced repetitive narrative around the maps and clarified the unique purpose of each figure group. The five figures remain as multi-panel figure groups because they correspond to distinct access dimensions: service landscape, modality/referral pathway, operating days, sex eligibility and consent requirements.	Map/figure narrative shortened and captions clarified to state each figure's distinct purpose; see Fig 1 lines 269-273, Fig 2 lines 303-307, Fig 3 lines 313-316, Fig 4 lines 323-325 and Fig 5 lines 328-330.
Figure shading makes all Lusaka appear uniformly high burden; use graduated symbols/proportional circles at ART-site level.	Addressed in the manuscript text and figure captions. We revised the mapping description to state that linked ART-site TX_CURR should be visualized using proportional circles rather than uniform district shading.	Mapping description and Fig 1 caption revised to specify facility-level proportional circles for linked ART-site TX_CURR, not uniform district shading; see lines 220-229 and 269-273.

We hope the revised manuscript satisfactorily addresses the reviewers' and editor's comments. We thank the reviewers again for their constructive suggestions.

Sincerely,

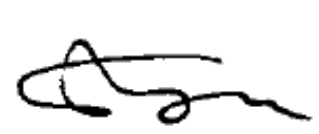

Carlos Muleya
School of Public Health, University of Zambia
Email: muleyacarlos@gmail.com | Tel: +260 979 863 766
