# Supplementary material for: Mapping mental health and psychosocial support service availability for adolescents and young people living with HIV in Lusaka and Shibuyunji districts, Zambia: Alignment with ART program burden (September 2024)
Source: PLOS Ment Health. 2026 Aug 25;3(8):e0000610. doi: 10.1371/journal.pmen.0000610 (PMC13505908; doi:10.1371/journal.pmen.0000610)
Supplement: Peer review history

# Peer review: Decision Letter 0

**DOI:** 10.1371/journal.pmen.0000610.r001

**Authors:** Karli Montague-Cardoso

17 Jul 2026

PMEN-D-26-00221

Mapping mental health and psychosocial support service availability for adolescents and young people living with HIV in Lusaka and Shibuyunji districts, Zambia: alignment with ART program burden (FY2024)

PLOS Mental Health

Dear Dr. Muleya,

Thank you for submitting your manuscript to PLOS Mental Health. I am very sorry for the delay. After careful consideration of the reviewer reports, we feel that your paper has merit but does not fully meet PLOS Mental Health’s publication criteria as it currently stands. Therefore, we invite you to submit a revised version of the manuscript that addresses the points raised during the review process. You can find all of the comments at the end of this email.

Please submit your revised manuscript by Aug 14 2026 11:59PM. If you will need more time than this to complete your revisions, please reply to this message or contact the journal office at mentalhealth@plos.org. When you're ready to submit your revision, log on to https://www.editorialmanager.com/pmen/ and select the 'Submissions Needing Revision' folder to locate your manuscript file.

Please include the following items when submitting your revised manuscript:

- A letter that responds to each point raised by the editor and reviewer(s). You should upload this letter as a separate file labeled 'Response to Reviewers'.
- A marked-up copy of your manuscript that highlights changes made to the original version. You should upload this as a separate file labeled 'Revised Manuscript with Track Changes'.
- An unmarked version of your revised paper without tracked changes. You should upload this as a separate file labeled 'Manuscript'.

Guidelines for resubmitting your figure files are available below the reviewer comments at the end of this letter.

As the corresponding author, your ORCID iD is verified in the submission system and will appear in the published article. PLOS supports the use of ORCID, and we encourage all coauthors to register for an ORCID iD and use it as well. Please encourage your coauthors to verify their ORCID iD within the submission system before final acceptance, as unverified ORCID iDs will not appear in the published article. *Only*  the individual author can complete the verification step; PLOS staff *cannot*  verify ORCID iDs on behalf of authors.

We look forward to receiving your revised manuscript.

Kind regards,

Karli Montague-Cardoso

Staff Editor

PLOS Mental Health

Journal Requirements:

1. Please ensure that your Ethics Statement is available in its entirety at the beginning of your Methods section, under a subheading 'Ethics Statement'.

2. Please amend your detailed Financial Disclosure statement. This is published with the article. It must therefore be completed in full sentences and contain the exact wording you wish to be published.

a. State the initials, alongside each funding source, of each author to receive each grant.

b. If any authors received a salary from any of your funders, please state which authors and which funders.

3. Please provide separate figure files in .tif or .eps format.

For more information about figure files please see our guidelines:  LINK

https://journals.plos.org/mentalhealth/s/figures

https://journals.plos.org/mentalhealth/s/figures#loc-file-requirements

4. We note that your Data Availability Statement is currently as follows: "De-identified facility mapping data and analytic code are available and have been shared as a supplementary file."

Please confirm at this time whether or not your submission contains all raw data required to replicate the results of your study. Authors must share the “minimal data set” for their submission. PLOS defines the minimal data set to consist of the data required to replicate all study findings reported in the article, as well as related metadata and methods (https://journals.plos.org/plosone/s/data-availability#loc-minimal-data-set-definition).

For example, authors should submit the following data:

- The values behind the means, standard deviations and other measures reported;

- The values used to build graphs;

- The points extracted from images for analysis.

Authors do not need to submit their entire data set if only a portion of the data was used in the reported study.

If your submission does not contain these data, please either upload them as Supporting Information files or deposit them to a stable, public repository and provide us with the relevant URLs, DOIs, or accession numbers. For a list of recommended repositories, please see https://journals.plos.org/plosone/s/recommended-repositories.

If there are ethical or legal restrictions on sharing a de-identified data set, please explain them in detail (e.g., data contain potentially sensitive information, data are owned by a third-party organization, etc.) and who has imposed them (e.g., an ethics committee). Please also provide contact information for a data access committee, ethics committee, or other institutional body to which data requests may be sent. If data are owned by a third party, please indicate how others may request data access.

If the reviewer comments include a recommendation to cite specific previously published works, please review and evaluate these publications to determine whether they are relevant and should be cited. There is no requirement to cite these works unless the editor has indicated otherwise.

Please review your reference list to ensure that it is complete and correct. If you have cited papers that have been retracted, please include the rationale for doing so in the manuscript text, or remove these references and replace them with relevant current references. Any changes to the reference list should be mentioned in the rebuttal letter that accompanies your revised manuscript. If you need to cite a retracted article, indicate the article’s retracted status in the References list and also include a citation and full reference for the retraction notice.

Additional Editor Comments (if provided):

[Note: HTML markup is below. Please do not edit.]

Reviewers' comments:

Reviewer's Responses to Questions

**Comments to the Author**

1. Does this manuscript meet PLOS Mental Health’s publication criteria?

Reviewer #1: Yes

Reviewer #2: Yes

**********

2. Has the statistical analysis been performed appropriately and rigorously?

Reviewer #1: Yes

Reviewer #2: Yes

**********

3. Have the authors made all data underlying the findings in their manuscript fully available (please refer to the Data Availability Statement at the start of the manuscript PDF file)?

The PLOS Data policy

Reviewer #1: Yes

Reviewer #2: Yes

**********

4. Is the manuscript presented in an intelligible fashion and written in standard English?

Reviewer #1: Yes

Reviewer #2: Yes

**********

Reviewer #1: This is an interesting article that addresses an important aspect of the management and care of adolescents living with HIV. It is widely recognised that psychosocial factors are crucial to the success of HIV treatment, and mapping the availability of mental health services is a necessary first step towards improving this aspect of healthcare. The study is generally well-conducted, with sound methodology; however, some revisions are required:

- For those unfamiliar with Zambia, it might be useful – either in the methodology section or, alternatively, in the introduction – to briefly explain how health services are organised, particularly for adolescents and with regard to HIV and mental health.

- Were any bibliographical references (e.g. WHO documents or tools) used in the development of the structured facility questionnaire? If so, these should be cited; if not, the process of developing the questionnaire should be explained in a little more detail.

- The author stated “Facilities were more densely distributed in Lusaka, while Shibuyunji facilities were more geographically dispersed”; It is unclear how geographical dispersion was assessed; this should be made explicit.

- The article’s bibliography is rather limited. It might be useful to expand it in the discussion by drawing comparisons with other sub-Saharan African countries, highlighting experiences of promoting mental health among adolescents living with HIV (see also doi:10.7189/jogh.14.04182) as well as the facilitators and barriers to accessing mental health services (see also doi:10.1186/s12889-025-22695-3).

- Authors should mention whether, and if so how, the matching criterion adopted – “using a prespecified rule (the first record in the cleaned dataset)” – may have introduced a bias, or whether this approach can instead be considered random

Reviewer #2: Overall, the manuscript is strong and methodologically innovative and appropriate. There is a strong rationale linking mental health to HIV outcomes and clearly establishes the need for service mapping. My recommendation is minor revisions based on the following suggestions:

Introduction:

-Explain why Lusaka and Shibuyunji were selected. Were these pilot districts, selected for burden, feasibility, or representativeness? This wasn’t justified.

-Please define TX_CURR at first mention. While this is a commonly used PEPFAR indicator, many readers outside HIV implementation may not be familiar with the term.

-The final paragraph introduces a future mobile application. I would mention it more cautiously because the present paper does not evaluate or develop the app, only provides foundational mapping data.

Methods:

-Explain the rationale for retaining the "first record" when multiple facilities matched a single ART site. Why is this preferable to another approach?

-Briefly discuss whether facility self-report could have introduced measurement bias.

-Even though this was mentioned in the Discussion, consider acknowledging in the Methods that excluding unmatched facilities from the linkage analyses could influence the interpretation of the burden-weighted results.

Results

-Some narrative is repetitive with the tables; consider shortening text where numbers are already presented.

Discussion:

-Be careful not to overinterpret statistical significance given the relatively small sample and exploratory nature of some comparisons

-Because service characteristics were self-reported by facilities rather than independently verified, consider noting the potential for information (self-report) bias as a study limitation.

-The section on the mobile application is more speculative than the data support. Rather than describing specific "low-regret functions," frame these as potential future applications informed by the mapping. The mapping may become outdated as services, hours, or referral pathways change, so the need for regular updating should be acknowledged.

-I would also emphasize that TX_CURR is a proxy for HIV program burden, not mental health burden, so conclusions should avoid implying direct alignment with mental health need.

Figures:

-Consider whether five maps are necessary. Several (particularly Figures 3–5) communicate very similar spatial patterns, so some could potentially be combined without losing important information.

-The district-level shading makes all of Lusaka appear to have uniformly high AYPLHIV burden, even though the paper reports that burden is actually concentrated in a relatively small number of ART sites (e.g., the top 10 sites account for 48.5% of the total burden). Using graduated symbols or proportional circles at the ART site level would better visualize where the highest concentrations of AYPLHIV are located within Lusaka, rather than implying that burden is evenly distributed across the district.

**********

what does this mean?). If published, this will include your full peer review and any attached files.

**Do you want your identity to be public for this peer review?**  If you choose “no”, your identity will remain anonymous but your review may still be made public.

For information about this choice, including consent withdrawal, please see our Privacy Policy

Reviewer #1: No

Reviewer #2: **Yes:** Charisse V. Ahmed

**********

[NOTE: If reviewer comments were submitted as an attachment file, they will be attached to this email and accessible via the submission site. Please log into your account, locate the manuscript record, and check for the action link "View Attachments". If this link does not appear, there are no attachment files.]

# Author comment: Author response to Decision Letter 1

**DOI:** 10.1371/journal.pmen.0000610.r002

23 Jul 2026

Attachment Submitted filename: 7.17.2026 PLOS_MH_Response_to_Reviewers.docx.pdf

# Editor report: Decision Letter 1

**DOI:** 10.1371/journal.pmen.0000610.r003

**Authors:** Karli Montague-Cardoso

5 Aug 2026

Mapping mental health and psychosocial support service availability for adolescents and young people living with HIV in Lusaka and Shibuyunji districts, Zambia: alignment with ART program burden (FY2024)

PMEN-D-26-00221R1

Dear Mr Muleya,

We are pleased to inform you that your manuscript 'Mapping mental health and psychosocial support service availability for adolescents and young people living with HIV in Lusaka and Shibuyunji districts, Zambia: alignment with ART program burden (FY2024)' has been provisionally accepted for publication in PLOS Mental Health.

Before your manuscript can be formally accepted you will need to complete some formatting changes, which you will receive in a follow up email. A member of our team will be in touch with a set of requests.

Please note that your manuscript will not be scheduled for publication until you have made the required changes, so a swift response is appreciated.

IMPORTANT: The editorial review process is now complete. PLOS will only permit corrections to spelling, formatting or significant scientific errors from this point onwards. Requests for major changes, or any which affect the scientific understanding of your work, will cause delays to the publication date of your manuscript.

If your institution or institutions have a press office, please notify them about your upcoming paper to help maximize its impact. If they'll be preparing press materials, please inform our press team as soon as possible -- no later than 48 hours after receiving the formal acceptance. Your manuscript will remain under strict press embargo until 2 pm Eastern Time on the date of publication. For more information, please contact mentalhealth@plos.org.

Thank you again for supporting Open Access publishing; we are looking forward to publishing your work in PLOS Mental Health.

Best regards,

Karli Montague-Cardoso

Staff Editor

PLOS Mental Health

***********************************************************

Reviewer Comments (if any, and for reference):
